# CT radiomics based model for differentiating malignant and benign small (≤20mm) solid pulmonary nodules

**DOI:** 10.3389/fonc.2025.1502932

**Published:** 2025-02-13

**Authors:** Jing-Xi Sun, Xuan-Xuan Zhou, Yan-Jin Yu, Ya-Ming Wei, Yi-Bing Shi, Qing-Song Xu, Shuang-Shuang Chen

**Affiliations:** ^1^ Department of Radiology, Xuzhou Central Hospital, Xuzhou, China; ^2^ Department of Information, Xuzhou Central Hospital, Xuzhou, China; ^3^ Department of Hospital Office, Xuzhou Central Hospital, Xuzhou, China; ^4^ Department of Taishan Community Service Center, Xuzhou Central Hospital, Xuzhou, China

**Keywords:** CT, radiomics, pulmonary nodule, small, prediction

## Abstract

**Background:**

Currently, the computed tomography (CT) radiomics-based models, which can evaluate small (≤ 20 mm) solid pulmonary nodules (SPNs) are lacking. This study aimed to develop a CT radiomics-based model that can differentiate between benign and malignant small SPNs.

**Methods:**

This study included patients with small SPNs between January 2019 and November 2021. The participants were then randomly categorized into training and testing cohorts with an 8:2 ratio. CT images of all the patients were analyzed to extract radiomics features. Furthermore, a radiomics scoring model was developed based on the features selected in the training group via univariate and multivariate logistic regression analyses. The testing cohort was then used to validate the developed predictive model.

**Results:**

This study included 210 patients, 168 in the training and 42 in the testing cohorts. Radiomics scores were ultimately calculated based on 9 selected CT radiomics features. Furthermore, traditional CT and clinical risk factors associated with SPNs included lobulation (P < 0.001), spiculation (P < 0.001), and a larger diameter (P < 0.001). The developed CT radiomics scoring model comprised of the following formula: X = -6.773 + 12.0705×radiomics score+2.5313×lobulation (present: 1; no present: 0)+3.1761×spiculation (present: 1; no present: 0)+0.3253×diameter. The area under the curve (AUC) values of the CT radiomics-based model, CT radiomics score, and clinicoradiological score were 0.957, 0.945, and 0.853, respectively, in the training cohort, while that of the testing cohort were 0.943, 0.916, and 0.816, respectively.

**Conclusions:**

The CT radiomics-based model designed in the present study offers valuable diagnostic accuracy in distinguishing benign and malignant SPNs.

## Introduction

Pulmonary nodules (PNs) are non-transparent lesions that are surrounded by the lung parenchyma and are not attributable to pleural effusion, atelectasis, or mediastinal lymphadenopathy (1–3). The two types of nodules include solid PNs (SPNs) and subsolid PNs, which require different management strategies as per the Fleischner guidelines (4). For > 8 mm SPNs, tissue sampling is advised (5–7). The thorough preoperative assessment of these SPNs is essential before the biopsy or video-assisted thoracoscopic surgery (VATS)-based wedge resection.

The benign and malignant SPNs are generally distinguished based on the clinical data, computed tomography (CT) findings, and tumor marker levels for each patient (8–10). Several efforts have been made to establish predictive models that can assess SPN malignancy risk by combining several predictors associated with malignant nodules (9), yielding models with 84% - 91% sensitivities and 74% - 80% specificities, along with the area under the curve (AUC) values between 0.83-0.89 (9). Therefore, more accurate predictive models are required for SPN assessment.

Radiomics has emerged as a novel approach for processing clinical images to extract high-dimensional quantitative data, thereby allowing for the characterization of tissue features undiagnosable (11, 12). Several radiomics-based models have also been designed to identify benign and malignant PNs based on their CT features (13–15). However, the assessment of SPNs is generally performed in a manner stratified based on nodule size (8, 16–18), with ≤ 20 mm SPNs being classified as small SPNs (8, 16, 17). The malignancy rates associated with different SPN sizes vary, suggesting that extant CT radiomics-based models may not be appropriate for evaluating small SPNs.

In this study, a CT radiomics-based model was designed to distinguish between benign and malignant small SPNs.

## Methods

This study was authorized by the Ethics Committee of Xuzhou Central Hospital, and the requirement of written informed consent was waived.

### Study design

This study enrolled small SPN patients consecutively from January 2019 to November 2021. The inclusion criteria included patients who indicated: (i) small SPNs ≤ 20 mm, (ii) a confirmed pathological SPN diagnosis after surgical resection, and (iii) a < 2-week interval between SPN detection and surgical resection. Patients were excluded if they had: (i) poor image quality; (ii) a history of malignancy, (iii) SPNs < 6 mm in diameter, or (iv) incomplete clinical data. Eligible patients were randomly assigned to training and testing cohorts at an 8:2 ratio.

### Clinical data

Clinical data were collected for all the patients including demographic factors (age, gender, smoking history), CT features (location, diameter, lobulation, spiculation, pleural retraction sign, CT bronchus sign, and calcification), and the levels of tumor markers [carcinoembryonic antigen (CEA), squamous cell carcinoma antigen (SCC), neuron-specific enolase (NSE), serum gastrin, cytokeratin 19 fragment (CYFRA21-1)]. The size of the SPNs was measured as the largest diameter on the axial CT images.

### CT images acquisition

A 64-row CT instrument (Brilliance 64 CT, Philips) was used for all CT imaging with the following settings: tube voltage = 120 kVp, tube current = 160-220 mAs, pitch = 0.97, and collimation = 0.6×128 mm. Images were reconstructed using a medium sharp (B50) reconstruction algorithm with a 1.0 - 1.25 mm thickness. The images of the lung (width = 1600 HU; level = -600 HU) and mediastinal (width = 450 HU; level = -50 HU) windows were analyzed. CT imaging features were assessed individually by two chest radiologists (JXS and XXZ) with 7 and 12 years of relevant experience, respectively, who were blinded to the pathological results for each patient.

### Feature extraction

A chief radiologist (YJY) with 7 years of experience manually segmented target 3D SPNs with the Radcloud platform (http://radcloud.cn) and remained blinded to patient pathological results. Further, the Radcloud platform was used for extracting the radiomics feature. Observer consistency was assessed using intra- and inter-class coefficient (ICC) values. Briefly, CT images from 20 randomly selected individuals in the training cohort were independently segmented by two radiologists (JXS and XXZ). Moreover, Reader 1 (JXS) repeated the segmentation of tumors from these 20 patients following a one-week interval. Repeatable features were regarded as those with an ICC ≥ 0.8, which were elected for subsequent evaluation. All remaining images were segmented by Reader 1 (JXS).

### Feature selection

Features with > 0.8 variances were identified with the variance threshold method for further analysis. Furthermore, based on the Selec-K-Best method analysis, features with a *p-value* of *< 0.05* were then retained for a final step in which, features associated with malignant SPNs were selected using a least absolute shrinkage and selection operator (LASSO) regression model. These characteristics were employed to formulate a radiomics signature, enabling the computation of radiomics scores for each respective patient.

### Development and validation of a CT radiomics-based model

To distinguish between malignant and benign small SPNs, a CT radiomics-based model was established. Briefly, univariate analysis (UA) and multivariate logistic regression analysis (MLRA) were carried out to select risk factors related to SPN malignancy in the training cohort. Then, a nomogram incorporating these risk factors and radiomics scores was established. Subsequently, the AUC values for receiver operating characteristic (ROC) curves were employed to assess the accuracy of the developed model. Moreover, the model was validated using data from the patients in the testing cohort.

### Clinical benefit assessment

The clinical application of the predictive model was assessed via a decision curve analysis of the training and testing cohorts.

### Statistical analyses

The statistical analysis was carried out using the SPSS 25.0 and R 4.1.2 software. Eligible patients were randomly assigned to training and testing cohorts at an 8:2 ratio using the Radcloud platform. The comparison of categorical data was carried out *via* Fisher’s exact test or χ^2^ test, while for continuous data, an independent sample t-test or Mann-Whitney U test was carried out. UA and MLRA were performed to identify factors that are associated with SPN malignancy. In the MLRA, particularly in UA, variables indicating a P-value < 0.1, were then selected. The comparison of AUC values was implemented using the DeLong test, and the predictive model’s performance was evaluated *via* calibration curves and the Hosmer-Lemeshow test. The statistical significance threshold was set as P < 0.05.

## Results

### Patients’ criteria

**Figure 1:**
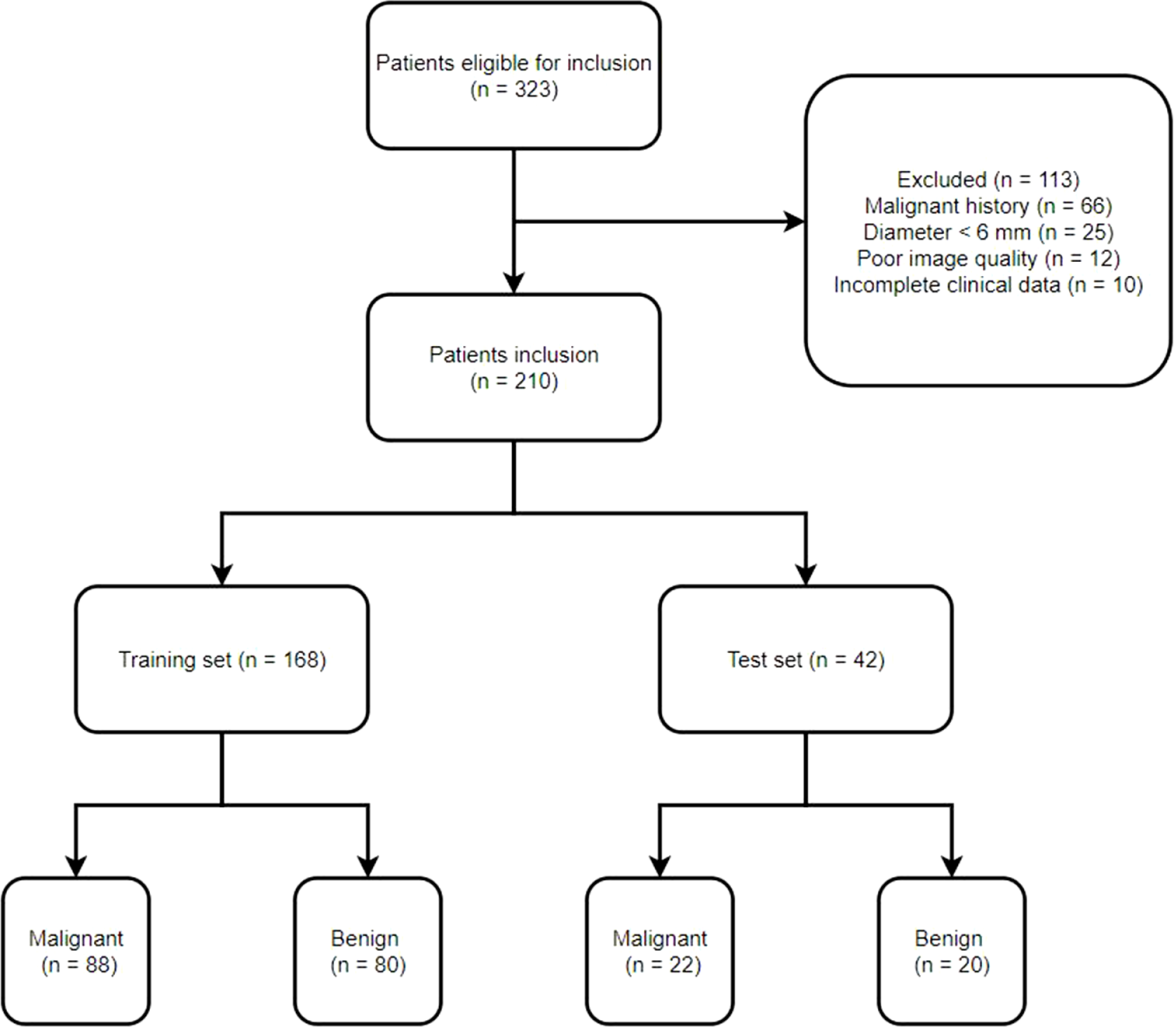
Study flowchart.

This study recruited 323 patients with small SPNs who underwent surgical resection procedures in our hospital from January 2019 to November 2021. Of these 323 patients, 210 were selected for further analyses (**Figure 1**). Each patient had a single SPN with a pathological diagnosis confirmed following surgical resection. All the participants were categorized into training (n = 168) and testing (n = 42) groups in an 8:2 ratio. **Table 1** indicates detailed information on the characteristics of selected patients.

**Table 1:** Baseline data of the patients.

		Training cohort (*n*=168)			Test cohort (*n*=42)			*p*-Inter
		Benign (*n*=80)	Malignant (*n*=88)	*p*-Intra	Benign (*n*=20)	Malignant (*n*=22)	*p*-Intra	
Clinical features								
Age (y)		57.88 ± 10.06	61.98 ± 9.60	0.008	52.60 ± 11.35	62.82 ± 10.25	0.004	<0.001
Gender [n (%)]	Male	33 (41.2)	47 (53.4)	0.155	7 (35.0)	9 (40.9)	0.94	0.148
	Female	47 (58.8)	41 (46.6)		13 (65.0)	13 (59.1)		
Smoker [n (%)]	No	60 (75.0)	66 (75.0)	1	13 (65.0)	14 (63.6)	1	1
	Yes	20 (25.0)	22 (25.0)		7 (35.0)	8 (36.4)		
CT imaging features								
Lobe location [n (%)]	Non-upper	38 (47.5)	49 (55.7)	0.365	9 (45.0)	13 (59.1)	0.546	0.223
	Upper	42 (52.5)	39 (44.3)		11 (55.0)	9 (40.9)		
Lobulation [n (%)]	No	53 (66.2)	33 (37.5)	<0.001	14 (70.0)	9 (40.9)	0.114	<0.001
	Yes	27 (33.8)	55 (62.5)		6 (30.0)	13 (59.1)		
Spiculation [n (%)]	No	48 (60.0)	33 (37.5)	0.006	11 (55.0)	9 (40.9)	0.546	0.004
	Yes	32 (40.0)	55 (62.5)		9 (45.0)	13 (59.1)		
Pleural retraction [n (%)]	No	41 (51.2)	34 (38.6)	0.137	13 (65.0)	8 (36.4)	0.122	0.031
	Yes	39 (48.8)	54 (61.4)		7 (35.0)	14 (63.6)		
CT bronchial sign [n (%)]	No	66 (82.5)	61 (69.3)	0.071	15 (75.0)	14 (63.6)	0.644	0.049
	Yes	14 (17.5)	27 (30.7)		5 (25.0)	8 (36.4)		
Diameter (mm)		9.76 ± 3.67	13.14 ± 4.79	<0.001	10.25 ± 4.27	11.41 ± 4.33	0.388	<0.001
Calcification [n (%)]	No	71 (88.8)	88 (100.0)	0.004	15 (75.0)	21 (95.5)	0.147	0.001
	Yes	9 (11.2)	0 (0.0)		5 (25.0)	1 (4.5)		
Tumor marker tests								
CEA (μg/L)		2.21 ± 1.34	2.42 ± 1.54	0.345	2.09 ± 0.91	2.51 ± 2.02	0.398	0.213
NSE (ng/ml)		13.02 ± 3.23	13.12 ± 3.27	0.848	13.15 ± 3.47	12.35 ± 3.60	0.47	0.857
SCC (μg/L)		1.53 ± 0.76	1.86 ± 0.81	0.321	1.86 ± 1.03	1.61 ± 0.71	0.373	0.43
Cyfra21-1 (ng/ml)		2.72 ± 1.18	2.55 ± 1.05	0.333	2.30 ± 0.93	3.00 ± 1.82	0.132	0.972

CEA, Carcinoembryonic antigen; CT, Computed tomography; NSE, Neuronspecifc enolase; SCC, Squamous cell carcinoma antigen.

### Feature selection and radiomics scoring

Initial analyses identified 1409 radiomics features. Then, to develop a radiomics score, a stepwise process was then employed (**Supplementary Table S1**), which revealed 9 features for radiomics score calculation (**Supplementary Table S2**). Coefficient values for all features as well as the mean square error for the combined sequences are presented in **Supplementary Figure S1**.

### Identification of malignancy-related clinicoradiological factors

The clinicoradiological features associated with malignant SPNs were assessed in the training cohort. The data revealed that the training cohort comprised 88 and 80 malignant and benign SPNs patients, respectively. UA identified older age (P = 0.01), lobulation (P < 0.001), spiculation (P < 0.001), and larger SPN diameter (P < 0.001) as being associated with a risk of SPN malignancy. Furthermore, MLRA confirmed that lobulation (P < 0.001), spiculation (P < 0.001), and larger SPN diameter (P < 0.001) were associated with a greater risk of SPN malignancy (**Table 2**).

**Table 2:** Predictors of malignancy in the training cohort (malignancy: 88/benign: 80).

	Univariate analysis			Multivariate analysis		
	OR	95% CI	p-value	OR	95% CI	p-value
Age	0.61	0.33-1.13	0.12			
Gender	1.04	1.01-1.08	**0.01**	1.02	0.99-1.06	0.2
Smoker	1	0.5-2.01	1			
Non-upper lobe	0.72	0.39-1.32	0.29			
Lobulation	3.27	1.74-6.16	**<0.001**	2.84	1.4-5.76	**<0.001**
Spiculation	2.5	1.34-4.65	**<0.001**	3.13	1.52-6.43	**<0.001**
Pleural retraction	1.67	0.9-3.08	0.1			
CT bronchial sign	2.09	1-4.34	0.05			
Diameter	1.2	1.11-1.3	**<0.001**	1.19	1.09-1.3	**<0.001**
Calcification	0	0-Inf	0.98			
CEA	1.11	0.89-1.38	0.35			
NSE	1.01	0.92-1.11	0.85			
SCC	1.11	0.88-1.38	0.38			
Cyfra21-1	0.87	0.66-1.15	0.33			

CEA, Carcinoembryonic antigen; CT, Computed tomography; NSE, Neuronspecifc enolase; SCC, Squamous cell carcinoma antigen.

Bold value means the statistical significance.

### Predictive model development

**Figure 2:**
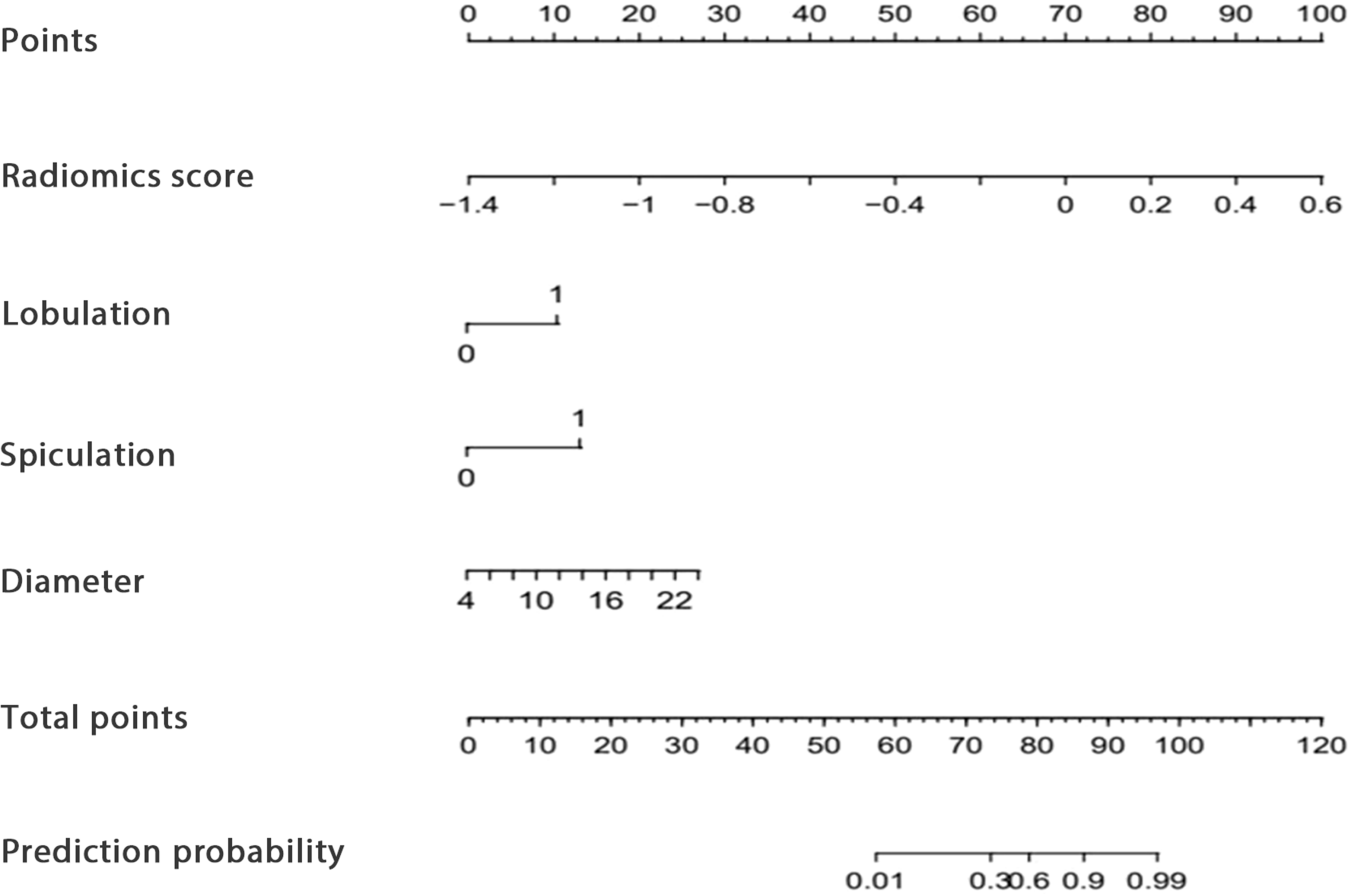
The developed CT radiomics model-based nomogram.

**Figure 3:**
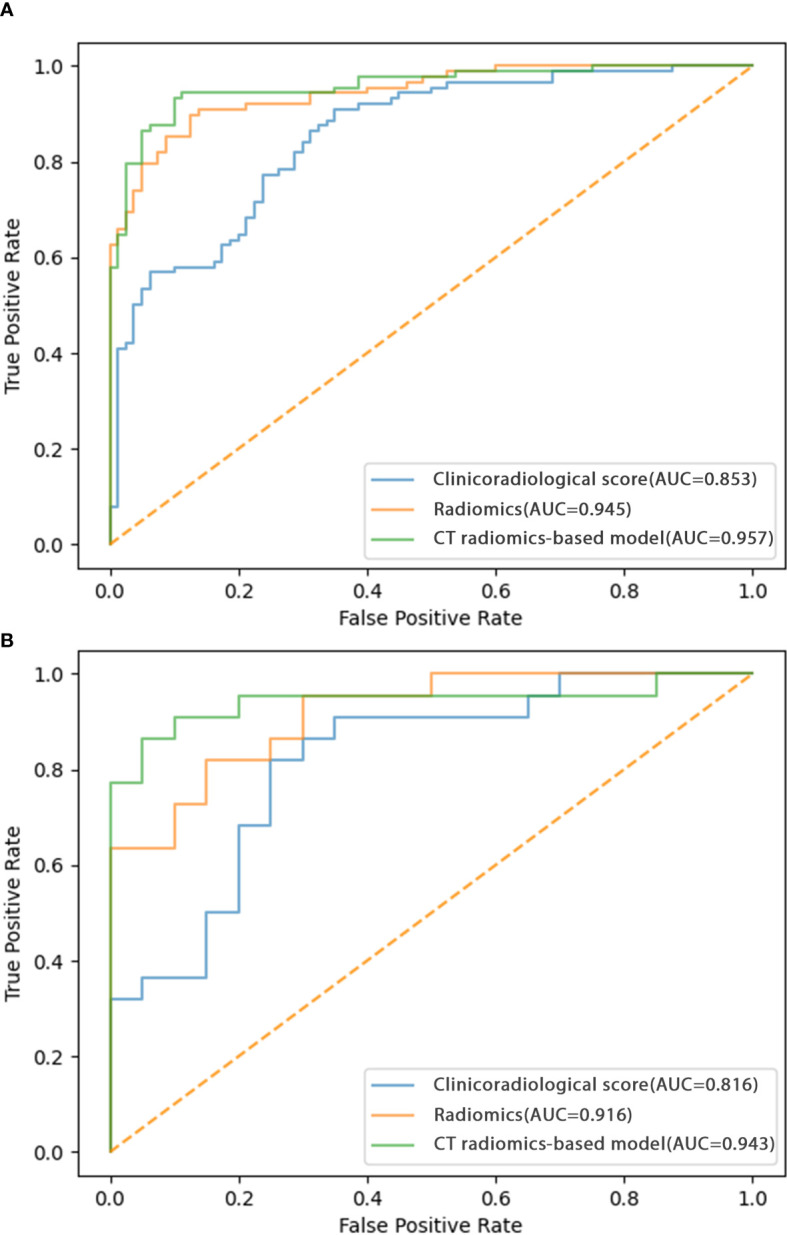
ROC curves corresponding to the CT radiomics-based model, CT radiomics score, and clinicoradiological score in the **(A)** training and **(B)** testing cohorts.

The identified clinicoradiological factors and radiomics score were used to establish a predictive model with the following formula: X = -6.773 + 12.0705×radiomics score+2.5313×lobulation (present: 1; no present: 0)+3.1761×spiculation (present: 1; no present: 0)+0.3253×diameter. A nomogram was also developed with this CT radiomics-based model (**Figure 2**). Furthermore, individual clinicoradiological and radiomics score models were also developed. Sensitivity, specificity, accuracy, and AUC measurements for these models are presented in **Table 3**. AUC values for the CT radiomics-based model, CT radiomics score, and clinicoradiological score were 0.957, 0.945, and 0.853 (**Figure 3A**). Moreover, the AUC values of the CT radiomics-based model were significantly higher those for both CT radiomics scores (P = 0.035) and clinicoradiological scores (P = 0.021).

**Table 3:** The diagnostic performance of each model.

Models	Cohorts	AUC (95%CI)	Accuracy	Sensitivity	Specificity
Clinicoradiologic model	Training	0.853 (0.799-0.897)	0.851	0.864	0.838
	Test	0.816 (0.620-0.842)	0.762	0.773	0.750
Radiomics score model	Training	0.945 (0.914-0.968)	0.887	0.898	0.875
	Test	0.916 (0.764-0.935)	0.810	0.818	0.800
CT radiomics based model	Training	0.957 (0.931-0.979)	0.911	0.920	0.900
	Test	0.943 (0.822-0.975)	0.857	0.909	0.800

AUC, area under curve; CT, computed tomography.

### Model validation

The testing cohort comprised 42 patients, including 20 benign and 22 malignant SPN patients. Using the models developed above as well as the testing cohort data, the AUC values of the CT radiomics-based model, CT radiomics score, and clinicoradiological score were assessed as 0.943, 0.916, and 0.816, respectively (**Figure 3B**). The AUC of the CT radiomics-based model was significantly greater than the CT radiomics score (P = 0.043) and clinicoradiological score (P < 0.001).

### Analysis of model clinical benefit

**Figure 4:**
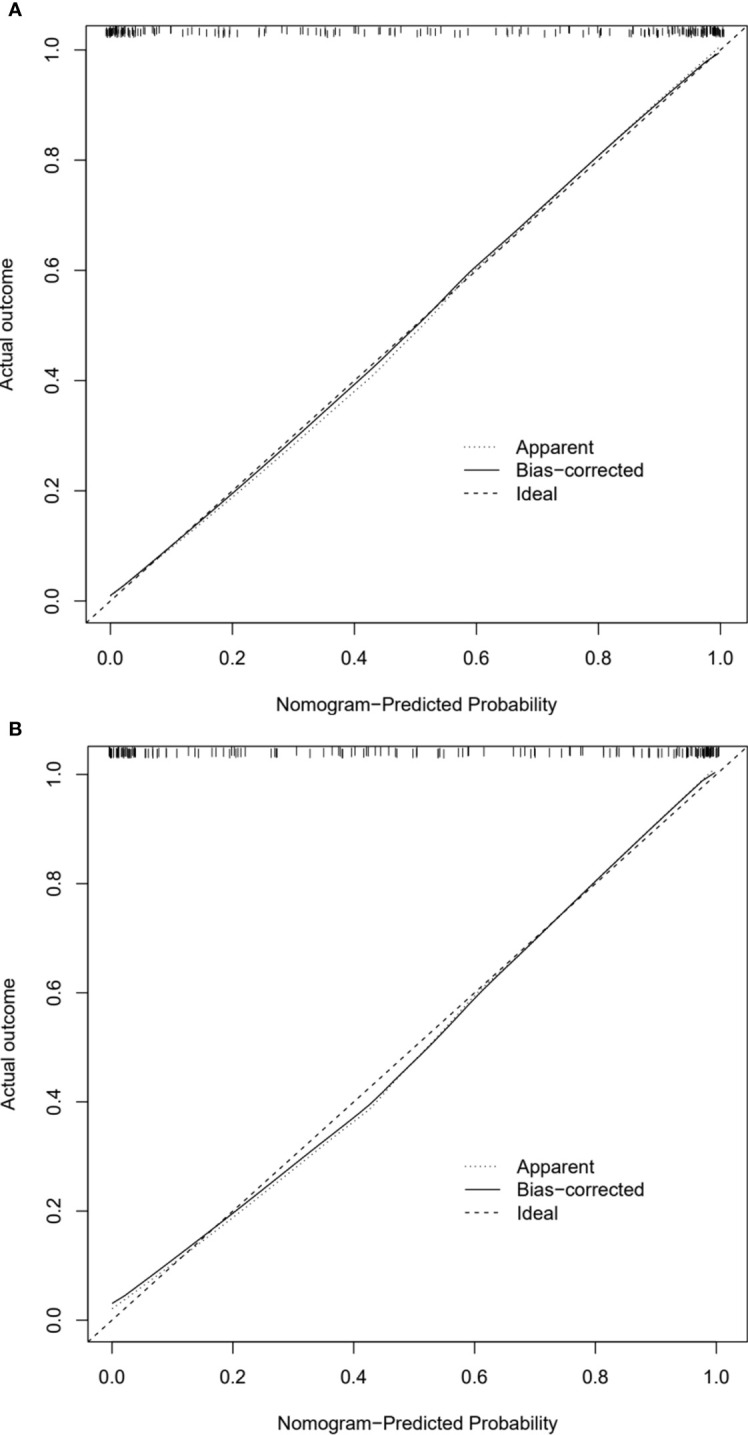
Calibration curves of CT radiomics-based model in the **(A)** training and **(B)** testing cohorts.

**Figure 5:**
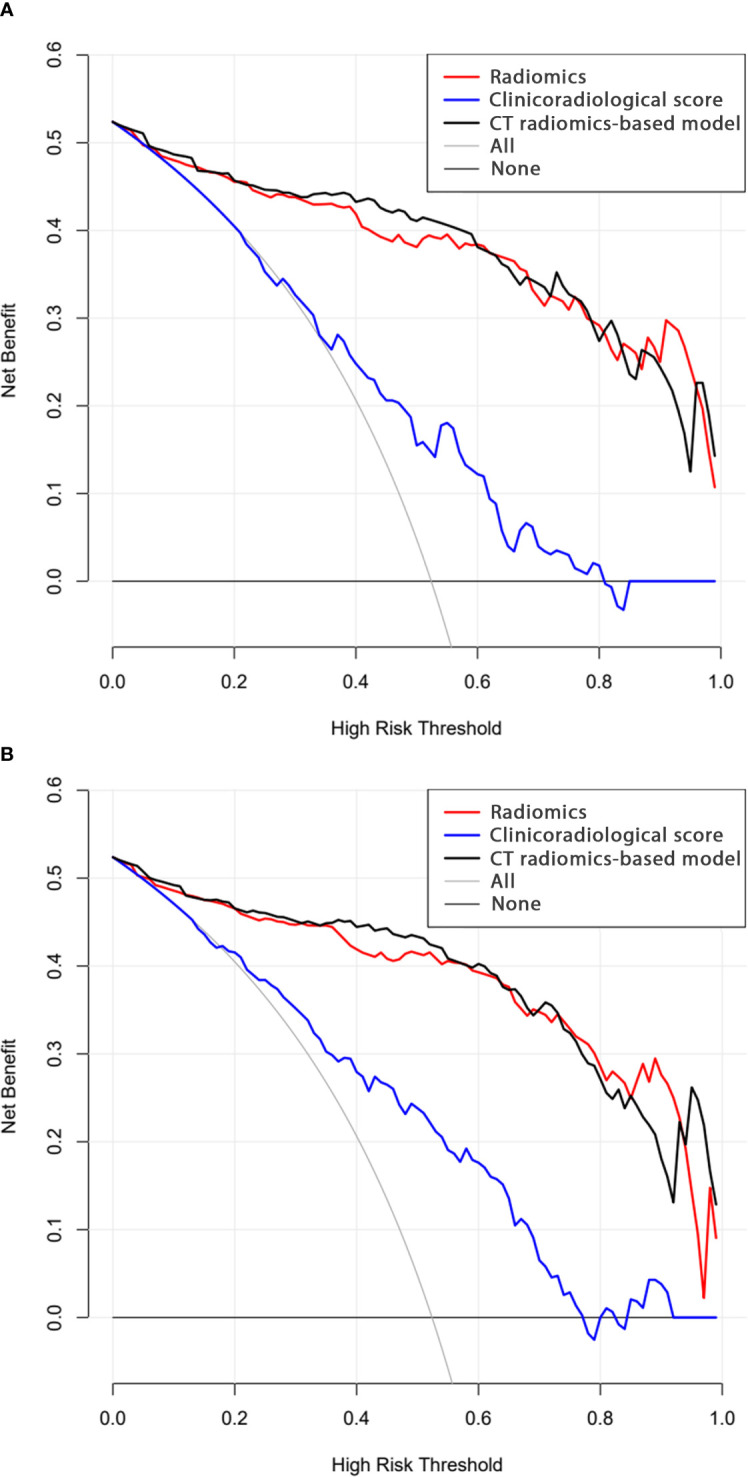
Decision curve analysis results for the **(A)** training and **(B)** testing cohorts.

In calibration curve analyses, the results predicted via the CT radiomics-based model and the actual results indicated good consistency in the training and testing cohorts (**Figures 4A, B**). Further, decision curves confirmed that the developed nomogram and associated predictive model yielded a net benefit in both cohorts with a risk threshold > 0 (**Figures 5A, B**).

## Discussion

Accurately diagnosing malignant SPNs is vital for the effective detection and management of lung cancer. Although the CT follow-up and longitudinal evaluation are often required for the SPNs, the follow-up for the high-risk SPNs may sometimes increase the risk of tumor growth. The Fleischner Society guidelines also recommended that the high-risk SPNs required tissue sampling (4). The predictive model is important because it can provide a comprehensive analysis for the SPNs and it can help us to make the next decision for CT follow-up or tissue sampling. Although various models have been designed to distinguish SPNs that are benign and malignant based on certain biomarkers (19–21), it is necessary to further stratify these analyses according to SPN size due to the high degree of variability in the malignancy rates of SPNs with different sizes (22). Some specific predictive models have been designed to identify small SPNs (8, 19, 23), however, further studies are required focusing on incorporating radiomics data into these models.

Variables such as clinical and tumor morphological characteristics are often incorporated into clinicoradiological predictive models aimed at differentiating benign and malignant SPNs (8, 19). The most common CT features of malignant SPN include a larger diameter, lobulation, spiculation, and CT bronchial sign (8, 19, 23). This study developed a more traditional predictive model based on clinical and tumor CT findings, which yielded AUC values of 0.853 and 0.816 in the training and testing cohorts, respectively. These AUC values align well with previously studied predictive models for small SPNs (0.744-0.878) (8, 19). However, these traditional CT features fail to offer any insight into the detailed internal structural properties of target tumors. Moreover, the identification of these features is often based on the experience of the radiologists who evaluate patient imaging results, therefore, they are prone to a high risk of bias.

The radiomics method entails the processing of medical images to extract high-dimensional quantitative data. This technique can characterize tumor microscopic features related to cellular, molecular, or gene expression patterns. Several studies support the application of radiomics to the differential diagnosis and prognostic assessment of several tumor types (12–14).

Here, a CT radiomics-based model was developed that could distinguish between benign and malignant small SPNs. This model was based on a combination of the radiomics scores and the established clinical model. The model showed an AUC value higher than that of the clinical model in the training (0.957 *vs*. 0.853, P = 0.021) and testing (0.943 *vs*. 0.816, P < 0.001) cohorts. These data validate that the radiomics score to significantly improves diagnostic performance relative to that associated with traditional clinical and radiological findings. The resultant nomogram can generate a direct predictive score for each small SPN, with this score corresponding to a predicted probability that can aid in clinical decision-making efforts.

Predictive models developed to evaluate small SPNs in previous reports determined that CEA levels were significantly related to the risk of malignancy (8, 23). One meta-analysis demonstrated that CEA had good diagnostic performance when used to distinguish between benign and malignant PNs (24). However, in the present study, no relationship was observed between tumor marker levels and the malignancy status of small SPNs. These discrepant results may be attributable to sample size limitations.

There are some limitations to the present study. For one, as a retrospective study, there is a high risk of selective bias. Secondly, this was a single-center study, therefore, prospective multi-center validation is required. Thirdly, some patient data at baseline was not balanced between the training and testing cohorts, potentially contributing to a greater risk of bias. However, both cohorts exhibited similarly high AUC values exceeding 0.9, suggesting a high degree of stability for the predictive model. Finally, because a radiomics approach was employed in this study, the reproducibility of this analytical strategy and its potential for standardization are limited, constraining the potential clinical application of this model.

## Conclusions

In summary, this study established a CT radiomics-based model that indicated satisfactory diagnostic accuracy in distinguishing between benign and malignant small SPNs.

## Data Availability

The raw data supporting the conclusions of this article will be made available by the authors, without undue reservation.

## Supplementary material

Supplementary Figure 1
**(A)** The coefficients for each individual feature and **(B)** combined sequence mean square errors.
