# Supplementary material for: CT radiomics based model for differentiating malignant and benign small (≤20mm) solid pulmonary nodules
Source: Front Oncol. 2025 Feb 13;15:1502932. doi: 10.3389/fonc.2025.1502932 (PMC11864964; doi:10.3389/fonc.2025.1502932)
Supplement: Supplementary file 2 [file DataSheet2.doc]

Supplementary Table 1 Procedures of radiomics features selection.

| Features input | Intra-class correlation  coefficient（>0.8） | Variance threshold（>0.8） | SelectKBest (p<0.05) | LASSO |
| --- | --- | --- | --- | --- |
| 1409 | 1184 | 764 | 116 | 9 |

LASSO: least absolute shrinkage and selection operator.

Supplement Table 2 Radiomics features and their coefficients

| Features | Coefficients |
| --- | --- |
| wavelet-LHL_gldm_LowGrayLevelEmphasis | -0.072178453 |
| wavelet-HHL_glszm_ZonePercentage | -0.074472865 |
| gradient_firstorder_InterquartileRange | -0.05114019 |
| wavelet-HLH_ngtdm_Contrast | 0.088569598 |
| wavelet-LLH_glcm_Imc2 | -0.076637658 |
| wavelet-LLH_glrlm_LongRunHighGrayLevelEmphasis | 0.08466695 |
| wavelet-LLL_glszm_LargeAreaHighGrayLevelEmphasis | 0.033382397 |
| original_shape_Flatness | 0.037915862 |
| lbp-2D_firstorder_90Percentile | -0.114951175 |
