# Supplementary figures and images for: CT radiomics based model for differentiating malignant and benign small (≤20mm) solid pulmonary nodules
Source: Front Oncol. 2025 Feb 13;15:1502932. doi: 10.3389/fonc.2025.1502932 (PMC11864964; doi:10.3389/fonc.2025.1502932)

| 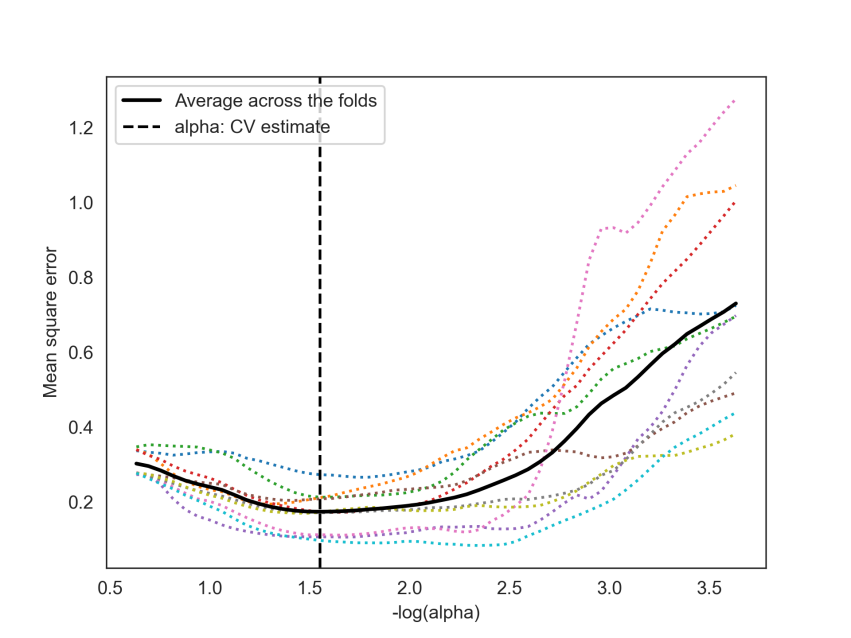 | 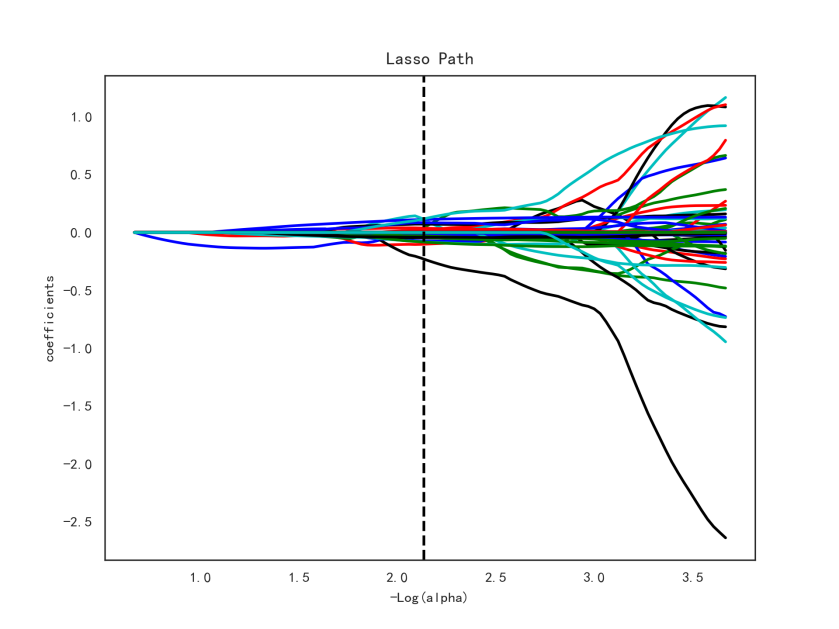 |
| --- | --- |
| A | B |

Supplement: Supplementary file 3 [file DataSheet3.docx]
